# Inulin aggravates colitis through gut microbiota modulation and MyD88/IL-18 signaling

**DOI:** 10.1080/19490976.2025.2570425

**Published:** 2025-10-24

**Authors:** Xu Zhao, Yadong Wang, Yanling Wang, Andrew T. Gewirtz, Jun Zou

**Affiliations:** Center for Inflammation, Immunity and Infection, Institute for Biomedical Sciences, Georgia State University, Atlanta, GA, USA

**Keywords:** Inulin, gut microbiota, innate immune cells, IL-18, maternal diet, colitis

## Abstract

Different types of dietary fibers exert distinct effects on gut microbiota and overall health, playing a complex role in disease-specific contexts. Here, we found that while supplementation with dietary soluble fiber inulin does not induce inflammation under steady-state conditions, it exacerbates intestinal inflammation in a DSS-induced colitis mouse model in a gut microbiota-dependent manner. Mechanistically, we found that inulin consumption increases the overall bacterial load with expansion of pro-colitis pathobiont, elevates levels of pro-inflammatory component flagellin, and reduces microbial diversity. These microbiota changes drove colitis aggravation via GR-1⁺ cell-mediated inflammation. Using NLRC4/TLR5- and MyD88-deficient mice, we identified microbiota-derived pro-inflammatory signals—particularly flagellin—as key mediators of this effect. Moreover, the exacerbation of colitis was abolished in IL-18 knockout mice, indicating a critical role for inflammasome signaling in mediating this response. Notably, maternal consumption of an inulin-enriched diet during lactation altered the offspring's gut microbiota and increased their susceptibility to DSS-induced colitis. Collectively, our findings suggest that inulin intake exacerbates DSS induced colitis through microbiota-mediated inflammation and MyD88/IL-18 signaling, with the effects persist in the offspring. These results underscore the importance of considering fiber type in disease-specific dietary interventions, supporting the idea that a more balanced intake of natural dietary fibers that foster a diverse and resilient gut microbiota may offer greater benefits for intestinal health.

## Introduction

Inflammatory bowel diseases (IBDs), such as ulcerative colitis and Crohn's disease, are long-term inflammatory conditions of the digestive tract that affect millions of people globally, with incidence rates continuing to rise at an alarming pace.^1^^,^^2^ Although over 200 risk-associated genomic loci have been discovered,^3^ it is now clear that environmental factors also play a pivotal role in disease onset and progression.^4^ Among these factors, gut microbiota has been widely recognized as a key contributor to IBD.^5^^,^^6^ Dysbiosis, characterized by an imbalance in the composition and function of gut microbial communities, has been consistently linked to increased intestinal inflammation and impaired mucosal barrier integrity in IBD patients.^7^^,^^8^ Altered microbial metabolism, increased levels of pro-inflammatory bacterial components, and reduced bacteria diversity have all been implicated in triggering immune dysregulation in the gut.^9^ Notably, both genetic predispositions and environmental influences—such as diet, antibiotic use, and early-life exposures—can shape the composition and functionality of the gut microbiota, thereby modulating host immune responses and inflammatory pathways^10^^,^^11^ Among these immune pathways, the MyD88/IL-18 signaling axis plays a critical role in regulating host–microbiota interactions and gut inflammation.^12^^,^^13^ Activation of MyD88-dependent pathways by microbial components can induce IL-18 production that can maintain barrier integrity.^14^ However, excessive IL18 signaling in intestinal epithelial cells (IECs) disrupts goblet cell maturation and exacerbates colitis.^15^ Consequently, dysregulation of this pathway is associated with increased susceptibility to colitis and altered microbiota composition.

Dietary fibers are common components of various foods, and some types can regulate gut microbiota by serving as a substrate for microbial fermentation,^16-18^ thereby influencing metabolic health and the development of conditions such as obesity, diabetes, and cardiovascular disease.^19^^,^^20^ Different dietary fibers possess distinct fermentation properties, which can lead to differential effects on gut microbiota composition and function.^21^^,^^22^ Consequently, while some dietary fibers have been shown to confer protective effects against intestinal inflammation, others can exacerbate colitis.^22^^,^^23^ Fermentable dietary fiber inulin, primarily extracted from chicory root and Jerusalem artichoke, is widely used in processed foods and dietary supplements and has been associated with metabolic health benefits,^24^ including improved lipid metabolism and glucose regulation.^25^^,^^26^ However, in the context of inflammatory bowel disease, the effects of inulin appear to be more complex.^27-29^ Our previous study described that inulin supplementation in composition defined diet exacerbates DSS-induced colitis in a mouse model,^27^ while the precise mechanisms by which inulin worsens colitis remain poorly understood.

Here, we aim to investigate the underlying mechanisms through which inulin aggravates DSS-induced colitis, with a particular focus on the role of gut microbiota. Our analysis of the gut microbiota revealed that inulin increases the overall microbial burden, promotes the expansion of pro-colitis pathobiont, and reduces microbial diversity. Although these microbial changes do not induce inflammation under normal conditions, they exacerbate intestinal inflammation in the context of DSS induced colitis through the activation of innate immune cells and the IL-18/MyD88-dependent signaling pathways.

## Results

### Inulin did not initial but aggravated DSS induced colitis

**Figure 1.**
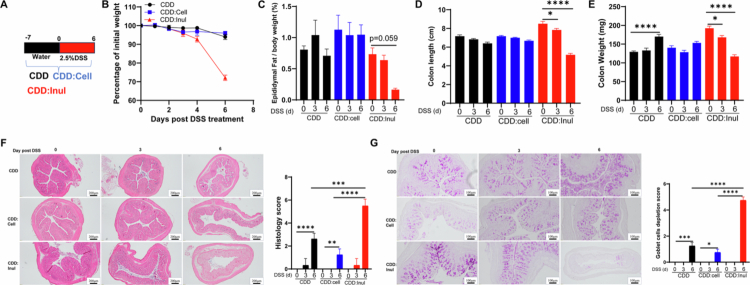
Inulin did not induce colitis but aggravated DSS-induced inflammation. Male C57Bl/6 mice were fed the specified diet for 7 d, followed by treatment with 2.5% DSS for 6 d, as illustrated in panel A. B. Body weight changes over the course of DSS treatment. C. Epididymal fat percentage relative to total body weight, measured and calculated at euthanasia time points. D. Colon length. E. Colon weight. F. H&E-stained colonic tissue sections with corresponding histopathological scores. G. Goblet cell staining and depletion analysis. Data are presented as mean ± SEM (*n* = 3–4 mice per group). All experiments were independently repeated 2–3 times, consistently producing similar results.

Our previous study demonstrated that a diet supplemented with the dietary fiber inulin could prevent HFD-induced obesity,^25^ but it exacerbated DSS-induced colitis, leading to greater body weight loss and bloody diarrhea at both low (50 g/kg) and medium (200 g/kg) doses.^27^ To further investigate the mechanisms underlying inulin-driven exacerbation of colitis, we selected the 200 g/kg dose of inulin because it closely approximates the fiber content of grain-based rodent chow (GBC), which contains approximately 15%–25% total fiber and has traditionally been used to maintain laboratory rodents.^30^ C57BL/6J wild-type mice were fed a compositionally defined diet (CDD) or a CDD supplemented with either cellulose—a non-fermentable fiber used as a control—or inulin (200 g/kg), a fermentable fiber, for 7 d (Figure 1A). Under this baseline condition, we assessed body weight, intestinal morphology, and inflammatory status. Mice fed the inulin-supplemented diet prior to colitis induction did not exhibit any adverse effects, as shown by stable body weight, preserved intestinal structure, and no increase in the percentage or number of innate immune cells, including neutrophils and macrophages, in the colon (Figure S1A–E). Consistent with these findings, analysis of published RNA-seq data^22^ from the colons of mice fed CDD or CDD:inulin diets (without DSS treatment) revealed no significant differences in the expression of inflammation-related genes under homeostatic conditions (data not shown). However, such inulin-fed mice showed extreme sensitivity to DSS-induced colitis, as indicated by rapid body weight loss (Figure 1B) and reduced fat percentage (Figure 1C). Morphometric and histopathological analyses further confirmed that inulin-fed mice developed more severe colitis than the control groups, as reflected by greater reductions in colon length and colon weight (Figures 1D,E; S1F), along with exacerbated colonic pathology—especially evident on day 6 post-DSS treatment (Figure 1F). Additionally, inflammation-associated goblet cell depletion was more pronounced in the inulin-fed group (Figure 1G), and barrier disruption—indicated by reduced tight junction protein staining—was more severe following DSS treatment (Figure S1G). Importantly, water consumption was comparable across all groups, ruling out increased DSS intake as the cause of heightened colitis susceptibility (Figure S1H). Food intake did not differ between groups without DSS administration; however, after DSS treatment, inulin-fed mice exhibited a significant reduction in food consumption, particularly on day 4 post-treatment (Figure S1I). This reduction was associated with elevated expression of the satiety-regulating hormone GLP-1 (Figure S1J). Together, these findings suggest that while inulin does not initiate colitis under steady-state conditions, it significantly aggravates DSS-induced colitis.

### Post-colitis intake of inulin impairs the recovery from DSS-induced colitis

**Figure 2.**
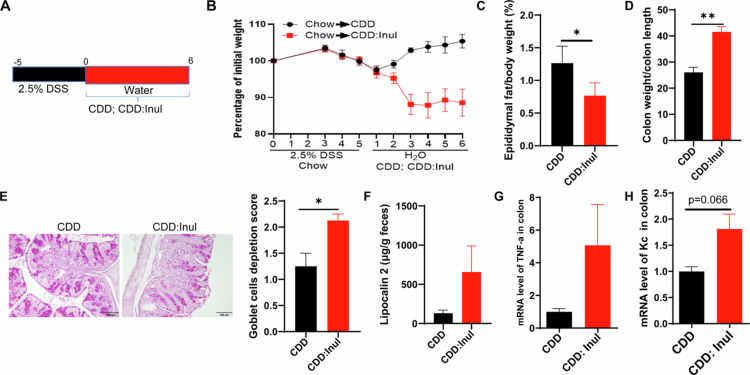
Inulin impairs recovery from DSS-induced colitis. A. C57Bl/6 mice were initially maintained on a chow diet and treated with 2.5% DSS for 5 d. DSS was then discontinued, and the mice were switched to a compositionally defined diet (CDD) or CDD supplemented with inulin (CDD: Inul). B. Body weight changes over time. C. Epididymal fat percentage relative to total body weight. D. Colon weight-to-length ratio. E. Goblet cell staining and depletion assessment. F. Fecal Lipocalin-2 levels measured by ELISA. G and H. Expression of TNF-α and Kc in colon tissue assessed by qRT-PCR. Data are shown as mean ± SEM (*n* = 4 mice per group).

To evaluate whether inulin consumption affects recovery from colitis, two groups of mice were first fed a grain-based chow (GBC) diet and treated with 2.5% DSS for 5 d to induce colitis (Figure 2A). Both groups exhibited comparable body weight loss during DSS administration (Figure 2B). Following DSS withdrawal, one group was transitioned to a compositionally defined diet (CDD), while the other received a CDD supplemented with inulin (CDD:Inul). After the dietary switch, mice in the CDD:Inul group continued to lose weight, whereas those in the CDD group began to recover body weight (Figure 2B), suggesting that inulin impairs post-colitis recovery. This delayed recovery in the inulin-fed group was further supported by a reduced fat mass percentage (Figure 2C), an increased colon weight-to-length ratio indicative of persistent low-grade inflammation (Figure 2D), and decreased goblet cell numbers in the colon (Figure 2E) measured at the end of experiment. Additionally, mice in the CDD:Inul group exhibited elevated fecal lipocalin-2 levels (Figure 2F) and increased expression of pro-inflammatory cytokines, including TNF-α and CXCL1 (KC) (Figure 2G,H). Collectively, these findings demonstrate that post-colitis inulin intake hinders recovery by sustaining or exacerbating intestinal inflammation.

### Inulin intake exacerbates DSS-induced colitis by enhancing Gr1 cells-mediated inflammation

**Figure 3.**
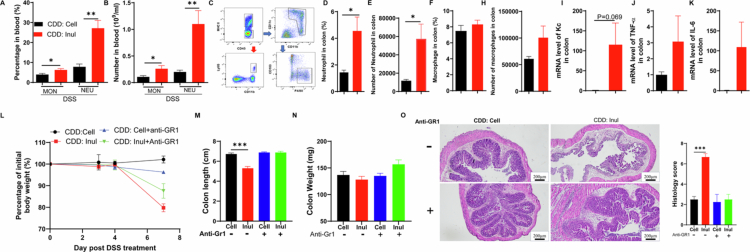
Inulin consumption worsens DSS-induced colitis by amplifying GR-1 cells driven inflammation. Following DSS treatment, blood samples were collected and analyzed using a hematology cell counter. A and B. The proportion and absolute count of monocytes (MON) and neutrophils (NEU). C–H. Flow cytometry analysis of neutrophils and macrophages in colonic tissue, with quantification of their percentage and number. I–K. qRT-PCR analysis of Kc, TNF-α and IL-6 expression in colon tissue. Wild-type mice subjected to DSS treatment received intraperitoneal injections of anti-Gr1 (200 µg/mouse) every other day. L. Body weight monitoring. M and N. Colon length and weight assessment post-euthanasia. O. Histological examination of colon tissue sections stained with H&E.

The observation that an inulin-enriched diet exacerbated colitis prompted us to investigate the associated inflammatory response. We first measured immune cell populations in the blood 6 d after DSS treatment using a hematology cell counter. The results showed that inulin-fed mice had a higher total white blood cell count compared with cellulose-fed controls, with a slight increase in the number of lymphocytes and a reduction in the percentage of lymphocytes (Figure S2A,B). Further analysis of neutrophils and monocytes, the two major types of white blood cells involved in inflammation, revealed that both the percentage and absolute number of neutrophils and monocytes were significantly elevated in the blood of inulin-fed mice (Figure 3A,B). To examine local inflammation, we analyzed neutrophil and macrophage populations in the colon by flow cytometry on day 6 post-DSS treatment (Figure 3C). Inulin-fed mice displayed increased infiltration of both macrophages and neutrophils in the colon relative to cellulose-fed controls, with neutrophils showing a particularly pronounced rise in both frequency and absolute number (Figure 3D–H). The increased recruitment of inflammatory innate immune cells in the colon was accompanied by elevated expression of pro-inflammatory cytokines, including KC, TNF-α and IL-6 (Figure 3I–K).

To access the contribution of neutrophils and inflammatory monocytes—cells that are recruited to the colon and can differentiate into macrophages—we used a well-established anti-Gr1 antibody to selectively deplete these populations.^31^ Flow cytometry confirmed that anti-Gr1 antibody treatment effectively reduced neutrophils and inflammatory monocytes (Figure S2C–E), and this depletion significantly mitigated the exacerbation of DSS-induced colitis caused by inulin, as evidenced by reduced weight loss (Figure 3L), preservation of colon length and mass (Figure 3M,N), and improved histopathological outcomes (Figure 3O). These protective effects were accompanied by a marked reduction in neutrophil infiltration in the colons of inulin-fed mice treated with the anti-Gr1 antibody (Figure S2F). These findings suggest that neutrophils and inflammatory monocytes, along with their derived macrophages, contribute to the intensified inflammatory response and worsened colitis observed with inulin supplementation.

### Impact of inulin-enriched diet on gut microbiota and bacterial translocation during DSS-induced colitis

**Figure 4.**
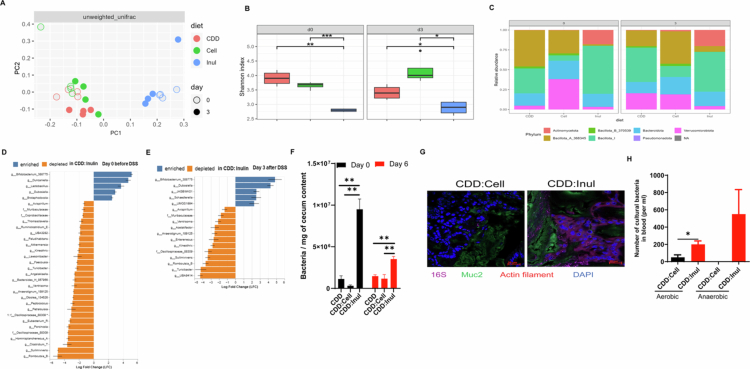
Influence of an inulin-supplemented diet on gut microbiota composition and bacterial translocation in DSS-induced colitis. A. Fecal microbiota profiling was performed using 16S rRNA sequencing, with overall microbial community structure represented by unweighted UniFrac PCoA analysis. B. Alpha diversity was measured using the Shannon index. C. Relative distribution of bacterial phyla. D and E. ANCOM-BC analysis reveals bacterial genera whose relative abundance was significantly enriched or depleted (*p*-adj < 0.01) in feces of inulin-fed mice compared to cellulose-fed mice, before (D) and after (E) DSS treatment. F. Total bacterial load in fecal DNA, collected before and after DSS exposure, was quantified via qPCR. G. Microbiota–mucus–epithelial interactions in colonic tissues from mice fed different diets were examined on day 4 post-DSS treatment using Carnoy's fixation, 16S rRNA FISH, and immunofluorescent staining. H. Blood samples obtained on day 6 post-DSS treatment were cultured on BHI agar under both aerobic and anaerobic conditions, and bacterial colony-forming units (CFU) were determined.

Previous studies, including our own, have demonstrated that dietary fiber can shape the gut microbiota.^25^^,^^26^^,^^32^ This prompted us to investigate key features of the gut microbiota—including relative composition, absolute abundance, and translocation—both before and after colitis induction in mice fed a compositionally defined diet (CDD) or CDD supplemented with either cellulose or inulin. Using 16S rRNA gene sequencing, we found that inulin significantly altered the overall gut microbiota composition, as evidenced by distinct clustering in principal coordinates analysis (PCoA) plots of β-diversity, both prior to and following 3 d of DSS treatment. These changes were evident using both Bray-Curtis distance (a quantitative measure of community dissimilarity) (Figure S2A) and Unweighted UniFrac distance (a qualitative measure incorporating phylogenetic relationships) (Figure 4A). Further analysis of α-diversity using Observed Features and the Shannon index revealed that inulin-fed mice had significantly reduced microbial diversity compared to those fed CDD or CDD supplemented with cellulose, both before and after DSS treatment. Specifically, the Observed Features were lower in the inulin group, indicating reduced species richness (Figure S2B), while the Shannon index—accounting for both richness and evenness—was also significantly lower (Figure 4B), suggesting a less diverse and more imbalanced microbiota. These changes in bacterial composition were further reflected by alterations in the relative abundance of different bacterial taxa at both the phylum and genus levels. At the phylum level, inulin-fed mice exhibited increased levels of Actinomycetota and Bacillota_I, while Verrucomicrobiota, Bacillota_A, and Bacillota_B were decreased (Figures 4C, S2C,D). At the genus level, while beneficial taxa such as Bifidobacteria were enriched, the pro-colitis bacterium Duncaniella was also significantly increased. Meanwhile, a greater number of genera were reduced in inulin-fed mice both before and after DSS treatment (Figure 4D,E), consistent with the overall decline in microbial diversity.

A recent publication demonstrated that the total quantity of fecal microbes plays a pivotal role in shaping gut microbiome composition and may be directly linked to disease outcomes.^33^ To assess absolute bacterial abundance, we performed quantitative PCR (qPCR) using a standard curve, which revealed that inulin supplementation significantly increased the total bacterial load both before and after DSS-induced colitis (Figure 4F). Breaching of the epithelial barrier by gut microbiota is a critical event that can trigger intestinal inflammation. To assess this, we used 16S FISH staining to examine bacterial localization during DSS-induced colitis and observed increased bacterial encroachment to the epithelium in inulin-fed mice (Figure 4G). This microbial encroachment was accompanied by enhanced bacterial translocation into the bloodstream, as evidenced by a higher number of culturable bacteria detected in blood of inulin-fed mice using both aerobic and anaerobic culture methods (Figure 4H). Together, these findings indicate that an inulin-enriched diet alters gut microbiota composition, increases bacterial load, and promotes microbial penetration of the gut barrier during DSS-induced colitis.

### Increased DSS induced colitis by inulin is dependent on gut microbiota

**Figure 5.**
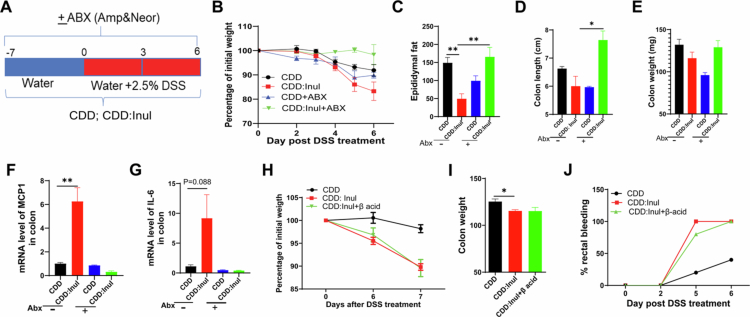
The exacerbation of DSS-induced colitis by inulin depends on gut microbiota. A. Mice were fed either a CDD or CDD:Inul diet, with or without an antibiotic cocktail containing ampicillin and neomycin. B. Following DSS treatment, body weight changes were tracked. C. epididymal fat mass was assessed. D and E. colon length and weight were measured. F&G. MCP-1 and IL-6 expression in colonic tissue was analyzed by qRT-PCR. In a separate experiment, mice were provided with CDD or CDD:Inul diets, with or without β-acid in in drinking water, during DSS treatment. H. Body weight was monitored. I and J. colon weight was recorded, and the percentage of rectal bleeding was calculated. Data are presented as mean ± SEM (*n* = 4 mice per group).

To determine whether inulin-induced changes in the gut microbiota contribute to exacerbated DSS-induced colitis, we treated mice fed either a CDD or CDD:Inulin diet with a broad-spectrum antibiotic cocktail, as previously described, to deplete gut microbiota.^25^ Both antibiotic-treated and untreated mice were then subjected to DSS-induced colitis (Figure 5A). Notably, antibiotic treatment reversed the difference in colitis severity between the CDD and CDD:Inulin groups. In inulin-fed mice without antibiotic, we observed greater body weight and epididymal fat loss, shortened colon length, reduced colon weight, and elevated levels of the inflammatory cytokines MCP-1 and IL-6. These effects were completely abolished by antibiotic treatment (Figure 5B–G). Together, these findings suggest that the colitis-promoting effect of inulin depends on the presence of gut microbiota.

One of the key metabolites generated during the gut microbiota fermentation of inulin is short-chain fatty acids (SCFAs).^26^ To test whether inulin-aggravated DSS-induced colitis is dependent on SCFA production, we treated one group of inulin-fed mice with beta acid, which we previously demonstrated significantly inhibits inulin fermentation and reduces SCFAs production.^25^ Inhibition of SCFAs production by beta acid did not prevent the exacerbation of DSS-induced colitis in inulin-fed mice, as indicated by comparable weight loss, colon weight, and rectal bleeding between the beta acid-treated and untreated inulin-fed groups (Figure 5H–J). These findings suggest that the colitis-promoting effect of inulin is not mediated by SCFAs produced during its microbial fermentation.

### MyD88-mediated signaling drives inulin-induced exacerbation of DSS-induced colitis

**Figure 6.**
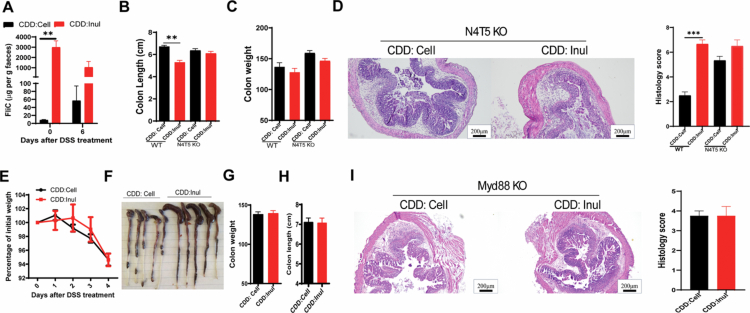
MyD88-dependent signaling contributes to inulin-induced worsening of DSS-induced colitis. A. Fecal samples were collected from mice before and after DSS treatment, resuspended in PBS at a concentration of 100 mg/ml, and centrifuged. The levels of flagellin in the supernatant were quantified using the HEK-TLR5 cell assay. B–D. N4T5 KO mice were fed either CDD:Cell or CDD:Inul diets and then subjected to DSS treatment for 7 d to induce colitis. Wild-type mice fed the same diets and treated with DSS (shown in Figure 3) served as controls. At the end of the experiment, B. colon length. C. colon weight was measured. D. Histological analysis of colonic tissue sections was performed using H&E staining. E–I. In a separate experiment, MyD88 KO mice were fed CDD:Cell or CDD:Inul diets and treated with DSS to induce colitis. E. Body weight changes were tracked. F–H. Gross morphology of the cecum and colon was assessed at the study endpoint, colon weight and length were recorded. I. Colonic tissue sections were examined histologically through H&E staining.

One feature of an inulin-enriched diet is its ability to promote the growth of specific bacteria, leading to an increased bacterial load in the intestine (Figure 4F). Along with the increased total bacterial count, there was a significant rise in the bacterial component flagellin (Figure 6A), accompanied by a parallel increase in anti-flagellin fecal IgA, particularly after DSS treatment in inulin-fed mice (Figure S3E). Because flagellin is a potent activator of innate immune cells through recognition by Toll-like receptor 5 (TLR5) and the NOD-like receptor family CARD domain-containing protein 4 (NLRC4), this increase in flagellin may activate innate signaling pathways that amplify inflammation and exacerbate colitis during DSS treatment. To test this possibility, we used N4T5 knockout mice, which lack the flagellin receptors, including NLRC4 and TLR5.^34^ Although colitis was still more severe in N4T5 knockout mice fed inulin compared to N4T5 KO mice fed cellulose, the extent of colitis observed in N4T5 knockout mice fed inulin versus cellulose was less severe than in wild-type mice fed inulin versus cellulose. This was supported by measurements of colon length (Figure 6B), colon weight (Figure 6C), and colon pathology (Figure 6D). This suggests that inulin-induced increases in bacterial component flagellin may play a partial role in the exacerbation of DSS induced colitis.

In addition to flagellin, inulin may also induce the production of other pro-inflammatory bacterial components that contribute to intestinal inflammation. To further investigate the role of innate signaling in inulin-aggravated colitis, MyD88 knockout mice—lacking MyD88, a central mediator of innate signaling^35^—were fed diets supplemented with either cellulose or inulin, and colitis was induced using DSS. In contrast to wild-type mice, MyD88 knockout mice fed either cellulose or inulin showed similar disease severity after DSS treatment, as indicated by comparable body weight loss, colon length, and colon weight (Figure 6E–H). Histopathological analysis further confirmed the absence of differences in the extent of colon damage between the MyD88 knockout fed with CDD:Cell and CDD: Inul. These results suggest that the exacerbation of colitis by inulin is dependent on MyD88-mediated signaling.

### IL-18 as a key molecule mediating inulin-aggravated DSS-induced colitis

**Figure 7.**
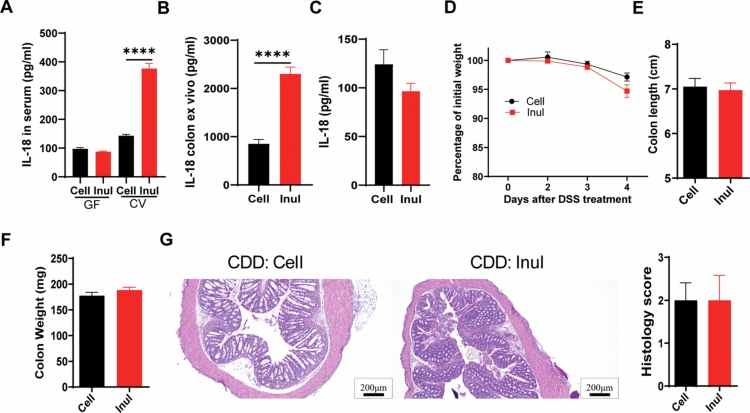
IL-18 as a mediator of inulin-exacerbated DSS-induced colitis. A. IL-18 levels in the serum of conventional wild-type (CV) and germ-free (GF) mice fed either cell or inulin enriched diet. B. IL-18 levels in ex vivo colonic tissue cultures from wild-type (WT) mice fed indicated diets. C. IL-18 levels in colon homogenate supernatants from MyD88 knockout (KO) mice fed the specified diets, measured by ELISA. D. IL-18 knockout (KO) mice were maintained on the indicated diets and subjected to DSS treatment, with body weight monitored throughout. E and F. Colon length and weight were assessed at the end of the experiment. G. Histological analysis of colonic tissue sections was conducted using H&E staining.

MyD88, a key adapter protein in innate immune signaling, regulates various downstream pathways that lead to cytokine production.^36^ Among these cytokines, interleukin-18 (IL-18) has been identified as a critical molecular mediator in the pathogenesis of colitis.^15^^,^^37^ To investigate whether the exacerbation of dextran sulfate sodium (DSS)-induced colitis by inulin supplementation via IL-18, we first evaluated the expression levels of IL-18 in serum during inulin feeding. Our results showed that inulin supplementation significantly upregulated IL-18 expression in the serum of conventional mice harboring gut microbiota. In contrast, IL-18 expression remained unchanged in germ-free mice, suggesting that the inulin-induced increase in IL-18 expression is dependent on the presence of gut microbiota (Figure 7A). Moreover, we found that inulin induced higher IL-18 levels in the colons of wild-type mice (Figure 7B). In contrast, MyD88 knockout mice—previously shown to develop similar severity of DSS-induced colitis regardless of diet—exhibited no significant differences in colonic IL-18 levels between dietary groups (Figure 7C). To clarify the functional role of IL-18 in inulin-aggravated DSS-induced colitis, we used IL-18 knockout (KO) mice that were cross-fostered with wild-type (WT) mice to normalize their gut microbiota. IL-18 KO mice fed with a cellulose enriched diet and those fed with an inulin-supplemented diet exhibited similar levels of DSS-induced colitis severity, as indicated by comparable weight loss, colon length, and colon weight (Figure 7D–F). Histological analysis further revealed that pathological features—including epithelial damage and increased inflammatory cell infiltration—were similar between the two diet groups (Figure 7G). Collectively, these findings demonstrate that IL-18 plays a pivotal role in mediating the exacerbation of DSS-induced colitis by inulin.

### Maternal inulin-enriched diet reduces offspring gut microbiota diversity and increases susceptibility to DSS-induced colitis

**Figure 8.**
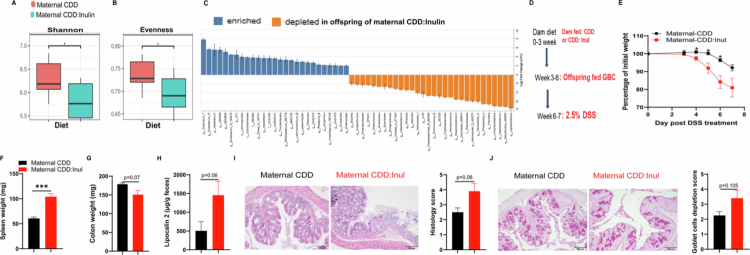
Maternal inulin-enriched diet reduces offspring gut microbiota diversity and increases susceptibility to DSS-induced colitis. A–B. Offspring from dams fed control (CDD) or inulin-enriched (CDD: Inulin) diets were weaned at 3 weeks of age and maintained on grain-based chow (GBC). Fecal samples were collected and analyzed by 16S rRNA sequencing, as described in a previous publication.^38^ Sequence data were evaluated for α-diversity using Shannon index (A) and evenness (B). C.ANCOM-BC analysis reveals bacterial genera whose relative abundance was significantly enriched or depleted (*p*-adj < 0.01, fold change larger than 2) in feces of offspring from dam that consumed inulin compared to those from dams that consumed cellulose. D. Offspring was maintained on GBC until 6 weeks of age, after which colitis was induced by DSS treatment. E. Body weight was tracked throughout DSS treatment. F and G. Spleen and colon weights were measured at the endpoint. H. Fecal lipocalin-2 levels were quantified by ELISA. I. Colonic tissue sections were assessed histologically by H&E staining. J. Goblet cell staining and depletion were evaluated. Data are presented as mean ± SEM (*n* = 4−5 mice per group).

Our previous study suggested that maternal diet not only influences the gut microbiota of dams consuming these diets but also alters the gut microbiota composition in their offspring, as shown by β-diversity analysis.^38^ Further analysis of α-diversity from the previously published 16S rRNA sequencing data^38^ showed that adult offspring from dams fed the CDD:Inulin diet had significantly reduced microbial diversity, as indicated by lower Shannon index (Figure 8A) and evenness values (Figure 8B), compared to offspring from CDD-fed dams. This reduction in microbial diversity was further supported by ANCOM-BC analysis, which revealed a broader depletion of bacterial genera accompanied by the expansion of certain taxa, including a notable rise in the pro-colitis bacterium Duncaniella (Figure 8C). This pattern mirrors the microbial changes observed in adult mice directly consuming an inulin-enriched diet.

These findings led us to hypothesize that a maternal inulin-enriched diet modifies the gut microbiota in offspring and increases their susceptibility to DSS-induced colitis. To test this hypothesis, we fed dams either a compositionally defined diet (CDD) or a CDD supplemented with inulin (CDD:Inulin) starting shortly after birth. Offspring was weaned at 3 weeks of age and subsequently maintained on a grain-based chow diet until 6 weeks of age, after which colitis was induced using DSS (Figure 8D). Offspring from inulin-fed dams exhibited significantly greater susceptibility to DSS-induced colitis than those from CDD-fed dams, as reflected by greater body weight loss, increased spleen weight, reduced colon weight, and elevated levels of the inflammatory marker fecal lipocalin-2 (LCN2) (Figure 8E–H). Histological analysis further confirmed more severe intestinal pathology in offspring from inulin-fed dams, including increased epithelial damage, crypt loss, and goblet cell depletion (Figure 8I–J). Collectively, these findings suggest that maternal inulin consumption during lactation reduces offspring gut microbiota diversity, and increases the abundance of pro-colitis bacteria, thereby heightening their susceptibility to DSS-induced colitis in adulthood.

## Discussion

The intestine harbors trillions of microorganisms with diverse functions, playing a crucial role in maintaining gut homeostasis and overall health.^39^^,^^40^ However, an imbalance in gut microbiota, or dysbiosis, has been implicated in the development and progression of inflammatory bowel disease (IBD).^41^^,^^42^ IBD, which includes Crohn's disease and ulcerative colitis, is widely believed to result from a dysregulated immune response to gut microbiota and their metabolites.^43^ Here, we demonstrate that a purified fiber inulin reshapes the gut microbiota by increasing the overall bacterial load and reducing microbial diversity, which heightens the risk of excessive activation of innate immune cells and exacerbates intestinal inflammation in DSS-induced colitis.

Our findings highlight the dual role of inulin in host health. While it exerts beneficial effects on metabolic health through mechanisms such as bacterial fermentation metabolites that stimulate intestinal GLP-1 and PYY secretion,^38^^,^^44^ it can also exacerbate intestinal inflammation in colitis.^27^ A healthy gut maintains a balance between beneficial and potentially harmful microbial signals.^45^ Under normal conditions, inulin appears to support this balance; however, in colitis, its effects may be detrimental. When fed inulin, the growth of beneficial bacteria is promoted, which is a hallmark of inulin's action. This results in an increased total bacterial load and elevated levels of pro-inflammatory bacterial components, which—if not properly regulated—can overstimulate the immune system. Meanwhile, these bacteria also ferment inulin to generate short-chain fatty acids (SCFAs), promoting IL-22 expression, reinforce intestinal barrier integrity, and stimulate the production of antimicrobial molecules that help confine bacteria within the gut lumen.^25^ However, in the context of colitis—where the intestinal barrier is already compromised—the increased bacterial load and elevated levels of microbial components, particularly flagellin, induced by inulin intake may intensify the activation of innate immune responses. In this vulnerable state, excessive immune activation may occur through NLRC4/TLR5/MyD88-dependent signaling pathways. This cascade, including inflammasome engagement, leads to the overproduction of the pro-inflammatory cytokine IL-18, a key mediator of colitis,^46^^,^^47^ which in turn drives tissue damage and exacerbates intestinal inflammation.

Gut microbial diversity is essential for maintaining a healthy gut ecosystem and plays a critical role in sustaining immune balance and promoting resilience against inflammatory diseases.^48^ While dietary fiber is generally recognized for enhancing microbial diversity,^49^ it is essential to recognize that dietary fibers vary in their chemical makeup and source, which affect their physical and chemical characteristics, such as water solubility, fermentability, and their interaction with the gut microbiome.^50^^,^^51^ Specifically, inulin, a type of dietary fiber, can differ depending on the plant source, with variations in chain length, degree of polymerization, and specific fermentability by gut microbes.^52^ In our current study, inulin—extracted from chicory root and commonly used as a processed food additive—was found to reduce microbial diversity in mouse model. We propose that, although inulin promotes beneficial bacteria such as Bifidobacteria in our current study, this selective stimulation may, hypothetically, come at the expense of overall microbial diversity by creating competitive advantages that limit the growth of other microbial populations. A reduction in microbial diversity is often associated with increased susceptibility to inflammatory bowel disease (IBD).^53^ Our study showed that offspring of dams fed an inulin-enriched diet during lactation exhibited a similar overall bacterial load, as previously described,^38^ but significantly reduced microbial diversity, as shown in our current study. This decline in diversity correlated with increased susceptibility to DSS-induced colitis in offspring of dams fed an inulin-enriched diet during lactation, suggesting that inheriting a low-diversity gut microbiota or early-life exposure to inulin may predispose offspring to an elevated risk of inflammatory bowel disease.

While inulin is known to promote the growth of beneficial bacteria such as Bifidobacteria, our study shows that inulin also promotes the expansion of Duncaniella muricolitica, a pathobiont associated with worsening colitis in mouse models.^54^ This finding suggests that inulin-induced enrichment of certain pathobionts may contribute to the heightened severity of colitis. Supporting this idea, studies have reported that a fiber-deficient diet can mitigate colitis by altering both the ecological niche and metabolic resources of gut pathobionts.^55^ Furthermore, in colitis-associated gut barrier dysfunction, bacterial cell wall components such as flagellin, which was elevated in inulin-fed mice, can translocate from the gut lumen into the epithelium or bloodstream, potentially contributing to tissue damage. Together, these observations underscore the complex and context-dependent effects of dietary inulin, highlighting that while it can support beneficial bacterial populations under healthy conditions, it may also create ecological conditions that favor the expansion of pathobionts or increase host susceptibility to inflammation in disease states.

In addition to fiber-specific properties such as chain length and fermentability, the host's gut microbiota composition—which varies among individuals and can be altered by disease—strongly influence the physiological effects of dietary fibers.^56^^,^^57^ For instance, while inulin is generally considered well tolerated and beneficial,^58^ some individuals with intestinal disorders may experience adverse outcomes.^59^ Similar variability has been reported in animal models: although some studies suggest that inulin supplementation alleviates colitis,^60^^,^^61^ our current findings, together with others,^29^ show that an inulin-enriched diet exacerbates DSS-induced colitis in a gut microbiota–dependent manner. These discrepancies likely stem from differences in inulin characteristics that shape microbial community structure, as well as host-specific baseline microbiota—for example, the presence of pathobionts that respond to inulin. Collectively, these observations highlight the importance of considering both inulin type and host-specific microbiota composition when recommending its intake, particularly for individuals at risk of inflammatory bowel disease (IBD).

Overall, we provided compelling evidence that the intake of purified, processed dietary fiber inulin reduced microbial diversity, likely by selectively promoting the growth of certain bacterial species while limiting the expansion of others, thereby increasing susceptibility to colitis. These findings highlight the potential advantage of natural fiber sources or balanced fiber mixtures, which support a more diverse and stable microbial community and may offer greater benefits for gut health than purified inulin alone. Emerging research on bioactive materials shows promise for the treatment of IBD,^62^^,^^63^ suggesting that combining tailored dietary fiber strategies with bioactive materials could provide a more effective approach for managing intestinal health. A limitation of this study is that it was conducted solely using a chemically induced colitis model; therefore, further investigation in spontaneous colitis models with genetically susceptible mice is needed to gain deeper insight into the effects of inulin on colitis. Additionally, more research is required to examine how inulin influences the gut microbiota in humans and its potential impact on intestinal health, particularly in the context of IBD.

## Material and methods

### Animal Models, diets, and treatments

C57BL/6 wild-type (WT) mice, IL-18 knockout mice, MyD88 knockout mice were obtained from Jackson Laboratory or bred at Georgia State University. The generation of TLR5−/−/NLRC4−/− mice has been described previously.^64^ All mice were maintained under approved animal protocols at Georgia State University (IACUC # A24025) and were fed a purified diet (Table 1, Research Diets, Inc.). At five weeks of age (or as specified), mice were placed on defined diets for one week before receiving drinking water containing 2.5% DSS (Lot # S7102, MP Biomedicals; MW: 36,000–50,000) to induce colitis. Body weight was monitored throughout the experiment. At the end of experiment, epididymal fat, colon length, and colon weight were measured, and tissues were collected for further analysis. For antibiotic treatment, mice were fed the designated compositionally defined diets along with drinking water containing ampicillin (1 g/L) and neomycin (0.5 g/L) for one week before DSS administration. To deplete neutrophils and inflammatory monocytes, wild-type C57BL/6 mice received intraperitoneal injections of 200 µg of anti-Gr1 antibody (clone RB6-8C5, Bio X Cell) every other day. Additionally, some mice received either a vehicle control (propylene glycol) or β-acid (20 ppm) in drinking water to inhibit short-chain fatty acid production, following previously established protocols,^25^ before being exposed to DSS to induce colitis.

### Assessment of food and water intake

To evaluate food and water intake, mice were housed in a clean cage with a pre-measured quantity of food and water. After 24 h, the remaining amounts were recorded, allowing for the calculation of daily consumption.

### Cytokine expression analysis by qRT-PCR

Colon tissue RNA was extracted using Trizol, following the manufacturer's instructions (Invitrogen). Quantification of IL-6, TNF-α, Kc and MCP-1 mRNA expression was performed using quantitative real-time PCR (qRT-PCR) with the Bio-Rad iScript™ One-Step RT-PCR Kit on a CFX96 system (Bio-Rad, Hercules, CA). The primers used for amplification were as follows: TNF-α: CGAGTGACAAGCCTGTAGCC, CATGCCGTTGGCCAGGA. Kc: TTGTGCGAAAAGAAGTGCAG and TACAAACACAGCCTCCCACA IL-6: GTGGCTAAGGACCAAGACC, GGTTTGCCGAGTAGACCTCA. MCP-1: GCTGGAGCATCCACGTGTT, TGGGATCATCTTGCTGGTGAA; 36B4 (Housekeeping gene): TCCAGGCTTTGGGCATCA, CTTTATTCAGCTGCACATCACTCAGA. Gene expression levels were normalized to the housekeeping gene 36B4, and relative transcript levels were calculated accordingly.

### Histological and immunofluorescence analyses of colon tissues using H&E, PAS, tight junction, and neutrophil staining

Colon tissues were fixed in 10% phosphate-buffered formalin at room temperature for a minimum of one week. After fixation, samples were transferred to 70% ethanol, embedded in paraffin, and sectioned into 5-µm thick slices for histological analysis using hematoxylin and eosin (H&E) staining. Pathological scoring was performed based on the extent of epithelial damage, crypt loss and immune cell infiltration. To visualize goblet cells, colon sections were deparaffinized, rehydrated, and stained using the Periodic acid-Schiff (PAS) method.

For assessment of intestinal barrier integrity, formalin-fixed colon tissues were sectioned into 5-µm slices, subjected to antigen retrieval, and incubated with an anti-tight junction protein 1 antibody (NBP1-85047). Sections were then stained with a fluorescent secondary antibody and mounted using a DAPI-containing medium.

For neutrophil staining, colonic tissues stored at −80 °C were embedded in OCT compound, snap-frozen, and sectioned at 5 µm. Frozen sections were stained with an anti-neutrophil elastase antibody (ab131260), followed by an Alexa Fluor 564-conjugated anti-rabbit IgG secondary antibody.

### ELISA

Fecal samples were homogenized in PBS at a concentration of 100 mg/ml, then centrifuged at 12,000 rpm for 10 min. The supernatant was collected and stored at −80 °C. The LCN-2 concentration in the supernatant was quantified using the DuoSet Mouse Lipocalin-2/NGAL ELISA kit (R&D Systems). For IL-18 measurement in colon tissue, two approaches were used: (1) Ex vivo colon cultures were prepared as previously described,^25^ and supernatants were collected following incubation; (2) alternatively, fresh colon tissues were homogenized in 500 µL of cold lysis buffer using homogenizer on ice. The homogenates were then centrifuged, and the resulting supernatants were collected for analysis. IL-18 concentrations were quantified using a commercial MBL mouse IL-18 ELISA kit, following the manufacturer's instructions.

### Hematology and flow cytometry analysis

Blood was collected from mice into heparinized tubes, and the immune cell composition was analyzed using a hematology analyzer (VetScan HM5; Abaxis, CA, USA). Colon lamina propria cells were isolated as previously described.^65^ To exclude dead cells, Alexa 430 NHS ester was used, and non-specific binding was blocked with 10 µg/ml of anti-CD16/anti-CD32 for 10 min in FACS buffer. For surface staining, cells were suspended in 0.1 ml of FACS buffer on ice and incubated for 20 min with fluorochrome-conjugated antibodies targeting CD45, MHC-II, CD11b, CD11c, F4/80, and Ly6G, to identify innate immune cell populations as previously detailed.^34^ After three washes, the stained cells were analyzed using a BD LSR II flow cytometer. Flow cytometry data were processed and analyzed with FlowJo software (TreeStar, Ashland, OR).

### Measurement of fecal flagellin and anti-flagellin IgA

Fecal samples and cecum content was homogenized in PBS at a concentration of 100 mg/ml and then centrifuged at 10,000 rpm for 10 min. The supernatant was collected and analyzed for flagellin levels using HEK-TLR5 cells as previously described,^66^ and flagellin specifical IgA by ELISA according to our previous described.^67^

### Bacterial quantification in feces

To determine the total bacterial load in feces, DNA was extracted from weighed fecal samples using the QIAamp DNA Stool MiniKit (Qiagen, Hilden, Germany). The extracted DNA was then analyzed by qPCR using the QuantiFast SYBR Green PCR kit (Bio-Rad, Hercules, CA) and universal 16S rRNA primers (8F: 5ʹ-AGAGTTTGATCCTGGCTCAG-3ʹ and 338 R: 5ʹ-CTGCTGCCTCCCGTAGGAGT-3ʹ). The bacterial count was calculated and expressed as the number of bacteria per mg of stool, based on a standard curve.

### Bacterial localization by FISH staining

Bacterial localization was assessed using 16S rRNA FISH staining, as previously described with some modifications.^25^ Colonic sections containing fecal material were fixed in methanol-Carnoy's solution (60% methanol, 30% chloroform, 10% glacial acetic acid) for at least 3 h at room temperature. Following fixation, the tissues were washed sequentially in methanol (2× for 30 min), ethanol (2× for 20 min), and xylene (2× for 20 min), then embedded in paraffin and sectioned at 5 μm thickness on glass slides. The tissue sections were dewaxed by preheating at 60 °C for 10 min, followed by incubation in xylene at 60 °C for 10 min, then xylene and 99.5% ethanol (both for 10 min). After deparaffinization, the sections were incubated overnight at 50 °C with the EUB338 probe (5ʹ-GCTGCCTCCCGTAGGAGT-3ʹ, labeled with Alexa 647 at the 5ʹ end) at a concentration of 10 μg/mL in hybridization buffer (20 mM Tris–HCl, pH 7.4, 0.9 M NaCl, 0.1% SDS, 20% formamide). To visualize mucus layers, mucin was stained using an anti-MUC2 antibody, while the epithelial cytoskeleton was counterstained with Phalloidin-Tetramethylrhodamine B isothiocyanate (Sigma, St. Louis, MO) at 1 μg/mL. The sections were then washed three times in PBS, allowed to dry, and mounted with DAPI-containing mounting medium for visualization.

### Gut microbiota composition analysis

To analyze the composition of the gut microbiota, 16S rRNA gene amplification was performed according to the Illumina 16S Metagenomic Sequencing Library preparation guide. Briefly, DNA was extracted and used to amplify the V4 region of the 16S rRNA gene with the following primers: 515FB: 5′TCGTCGGCAGCGTCAGATGTGTATAAGAGACAGG TGYCAGCMGCCGCGGTAA-3′ 806RB: 5′GTCTCGTGGGCTCGGAGATGTGTATAAGAGACAGGGACTACNVGGGTWTCTAAT-3′. These primers were designed with overhang Illumina adapters. PCR products were purified using Ampure XP magnetic beads (Agencourt) and verified for amplicon size using a High Sensitivity Chip on a Bioanalyzer. A second round of PCR was performed to attach dual indices and Illumina sequencing adapters with the Nextera XT Index kit. The products were quantified, and DNA was pooled in equimolar ratios. The pooled DNA was quantified again before sequencing on an Illumina MiSeq sequencer (paired-end reads, 2 × 250 base pairs) at the Georgia Institute of Technology Molecular Evolution Core (Atlanta, GA). Following sequencing, the data were demultiplexed, quality-filtered, denoised, merged, and chimera removed using the DADA2 plugin in Qiime2. Principal Coordinate Analysis (PCoA) was visualized using R (version 4.3.2). Taxonomy was assigned using the Greengenes2 16S rRNA gene database (version 2024.09, animal distal gut habitat). To identify bacterial phylum- and genus-level differences between groups, ANCOM-BC analysis was conducted (adjusted *p*-value < 0.01).

### Statistical analysis

Statistical significance was determined using an unpaired Student's *T*-test for comparisons between two groups or one-way ANOVA for comparisons among three or more groups. Differences between experimental groups were considered significant when *p ≤ 0.05, **p ≤ 0.01, ***p ≤ 0.001, or ****p ≤ 0.0001.

**Table 1.** Compositionally defined diet used in this study.

	D12450J		D13081109		D13081108	
	CDD		CDD: Cell		CDD: Inul	
Product #	gm%	kcal%	gm%	kcal%	gm%	kcal%
Protein	19	20	17	20	18	20
Carbohydrate	67	70	59	70	74	70
Fat	4	10	4	10	4	10
Total		100		100		100
kcal/gm	3.8		3.37		3.5	

Ingredient	gm	kcal	gm	kcal	gm	kcal
Casein	200	800	200	800	200	800
L-Cystine	3	12	3	12	3	12
Corn starch	506.2	2025	506.2	2025	456.2	1825
Maltodextrin 10	125	500	125	500	125	500
Sucrose	68.8	275	63.8	255	68.8	275
Cellulose, BW200	50	0	200	0	0	0
Inulin	0	0	0	0	200	200
Soybean Oil	25	225	25	225	25	225
Lard	20	180	20	180	20	180
Mineral Mix, S10026	10	0	10	0	10	0
DiCalcium phosphate	13	0	13	0	13	0
Calcium carbonate	5.5	0	5.5	0	5.5	0
Potassium citrate, 1 H2O	16.5	0	16.5	0	16.5	0
Vitamin mix, V10001	10	40	10	40	10	40
Choline bitartrate	2	0	2	0	2	0
FD&C Yellow Dye #5	0.04	0	0	0	0.025	0
FD&C Red Dye #40	0	0	0	0	0	0
FD&C Blue Dye #1	0.01	0	0.05	0	0.025	0
**Total**	**1055.1**	**4057**	**1205.05**	**4057**	**1155.1**	**4057**

## Supplementary Material

Supplementary material Figure S1. Inulin alone did not cause colitis but exacerbated DSS-induced inflammation. Male C57Bl/6 mice were maintained on the indicated diet for 7 d, followed by treatment with or without 2.5% DSS. A. Changes in body weight of without 2.5% DSS treatment over time. B–E. Flow cytometry analysis of colonic neutrophils and macrophages, including quantification of their proportions and absolute numbers. F. Macroscopic examination of colon morphology. G. Analysis of intestinal barrier integrity by staining for tight junction proteins. H–I. Monitoring of water and food intake with or without 2.5% DSS treatment. J. RT-PCR analysis of GCG expression in colonic tissue. Data are expressed as mean ± SEM (*n* = 3–4 mice per group).

Supplementary material Figure S1.

Supplementary material Figure S2.

Supplementary material Figure S3.

## Data Availability

The sequencing data are deposited at SRA database under accession number PRJNA1265231.
