# Supplementary material for: Inulin aggravates colitis through gut microbiota modulation and MyD88/IL-18 signaling
Source: Gut Microbes. 2025 Oct 24;17(1):2570425. doi: 10.1080/19490976.2025.2570425 (PMC12562683; doi:10.1080/19490976.2025.2570425)
Supplement: Supplementary material — Figure S1. Inulin alone did not cause colitis but exacerbated DSS-induced inflammation. Male C57Bl/6 mice were maintained on the indicated diet for 7 d, followed by treatment with or without 2.5% DSS. A. Changes in body weight of without 2.5% DSS treatment over time. B–E. Flow cytometry analysis of colonic neutrophils and macrophages, including quantification of their proportions and absolute numbers. F. Macroscopic examination of colon morphology. G. Analysis of intestinal barrier integrity by staining for tight junction proteins. H–I. Monitoring of water and food intake with or without 2.5% DSS treatment. J. RT-PCR analysis of GCG expression in colonic tissue. Data are expressed as mean ± SEM (n = 3–4 mice per group). [file KGMI_A_2570425_SM4513.docx]

**Figure S1. Inulin alone did not cause colitis but exacerbated DSS-induced inflammation.** Male C57Bl/6 mice were maintained on the indicated diet for 7 days, followed by treatment with or without 2.5% DSS. A. Changes in body weight of without 2.5% DSS treatment over time. B–E. Flow cytometry analysis of colonic neutrophils and macrophages, including quantification of their proportions and absolute numbers. F. Macroscopic examination of colon morphology. G. Analysis of intestinal barrier integrity by staining for tight junction proteins. H-I. Monitoring of water and food intake with or without 2.5% DSS treatment. J. RT-PCR analysis of GCG expression in colonic tissue. Data are expressed as mean ± SEM (n = 3–4 mice per group).

**Figure S2. Anti-Gr1 treatment depletes circulating myeloid cells and reduces neutrophil infiltration in the colon**. A&B. Following DSS treatment, blood samples were collected from mice fed the indicated diet and analyzed using a hematology cell counter to determine white blood cell and lymphocyte counts. C-E. Wildtype C57BL/6 mice were fed either CDD:Cell or CDD:Inul diets for 6 days, followed by injection of anti-Gr1 antibody. Blood monocytes and neutrophils were analyzed by flow cytometry before and 1 day after anti-Gr1 treatment. F. Colon tissue from CDD:Cell- or CDD:Inul-fed mice, with or without anti-Gr1 treatment, was collected after 7 days of DSS treatment and stained for neutrophils.

**Figure S3. Influence of an inulin-supplemented diet on gut microbiota composition in DSS-induced colitis.** A. Fecal microbiota profiling was performed using 16S rRNA sequencing, with overall microbial community structure represented through Bray-Curtis analysis. B. Alpha diversity was assessed using observed features (OTUs). C&D. ANCOM-BC analysis depicting changes in the relative abundance of bacterial phyla in mouse fecal samples before (C) and after (D) DSS treatment. E. Fecal flagellin-specific IgA was measured in fecal samples prior to DSS treatment and in cecal contents after 3 days of DSS treatment in mice fed the indicated diets.
