# Supplementary figures and images for: Inulin aggravates colitis through gut microbiota modulation and MyD88/IL-18 signaling
Source: Gut Microbes. 2025 Oct 24;17(1):2570425. doi: 10.1080/19490976.2025.2570425 (PMC12562683; doi:10.1080/19490976.2025.2570425)

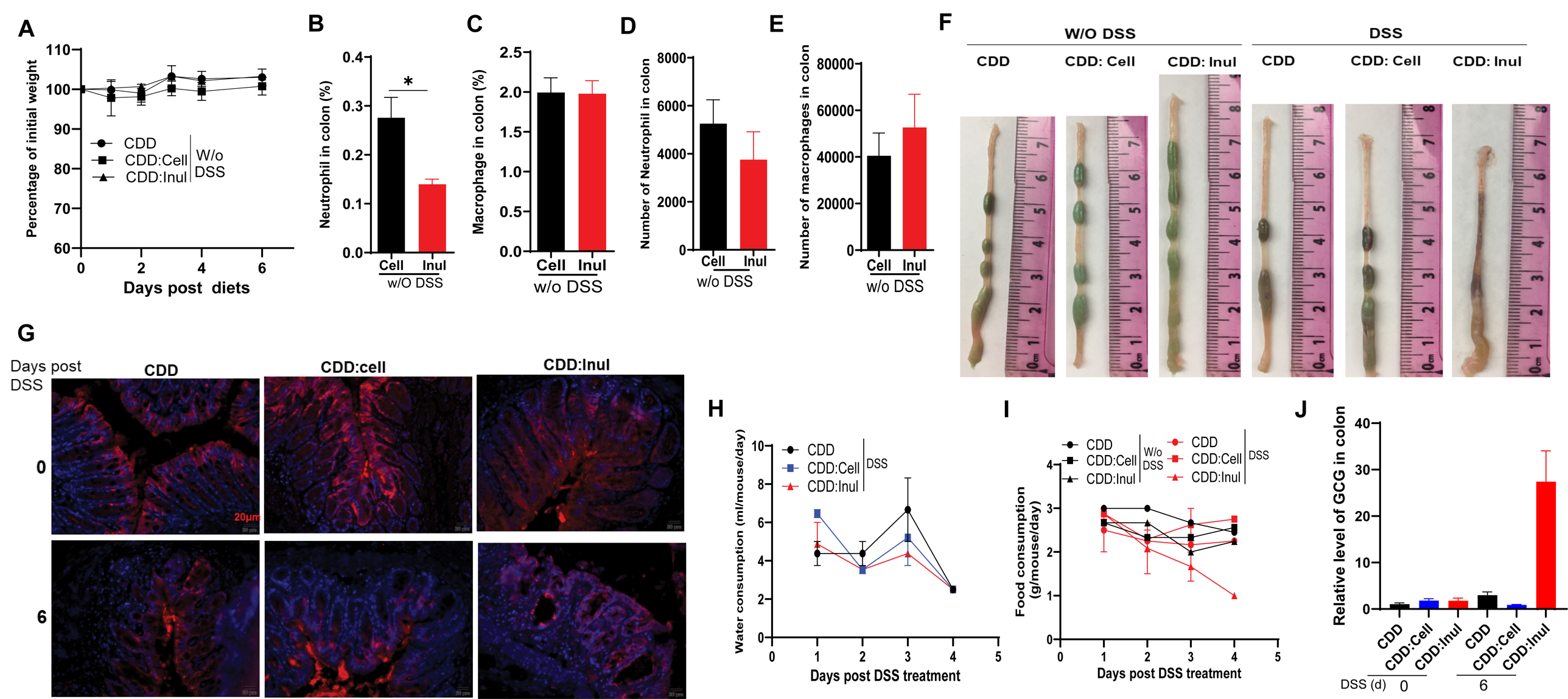

Supplement: Supplementary material — Figure S1. [file KGMI_A_2570425_SM4460.tif]

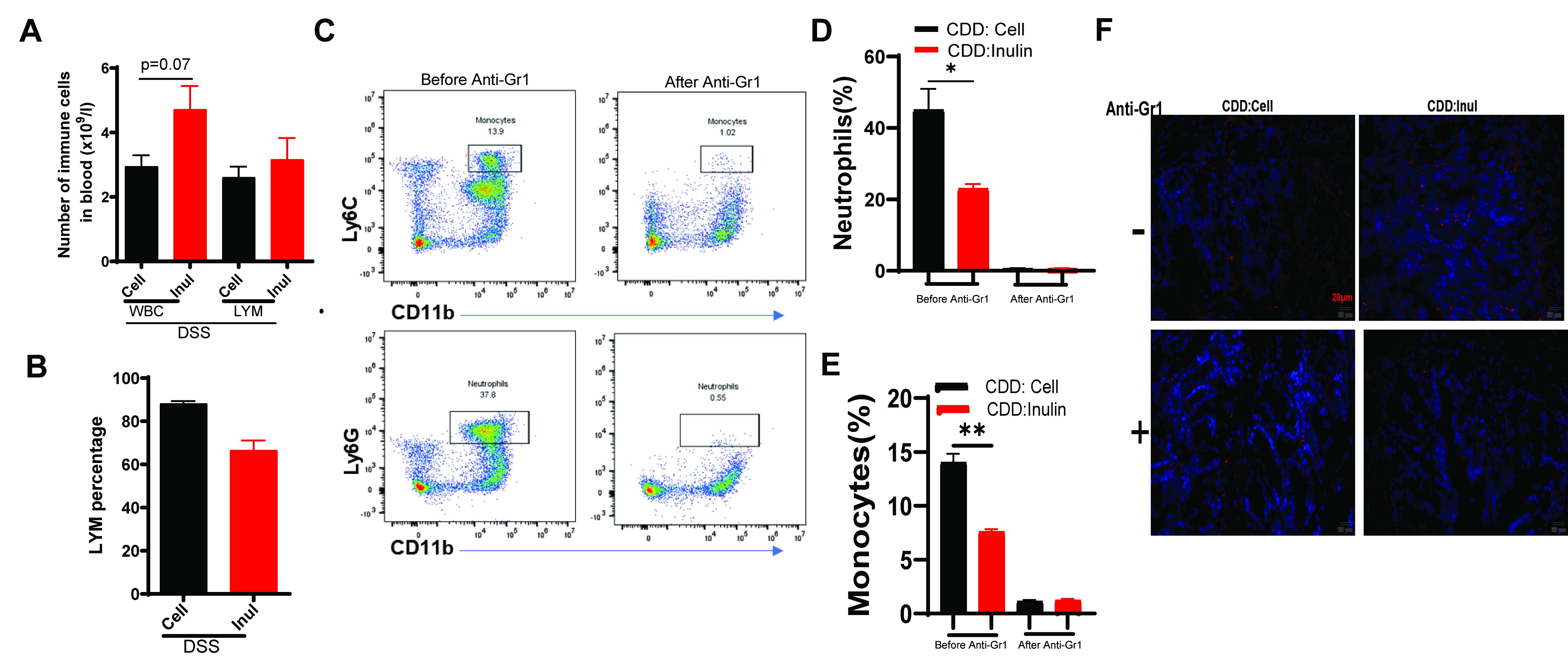

Supplement: Supplementary material — Figure S2. [file KGMI_A_2570425_SM4477.tif]

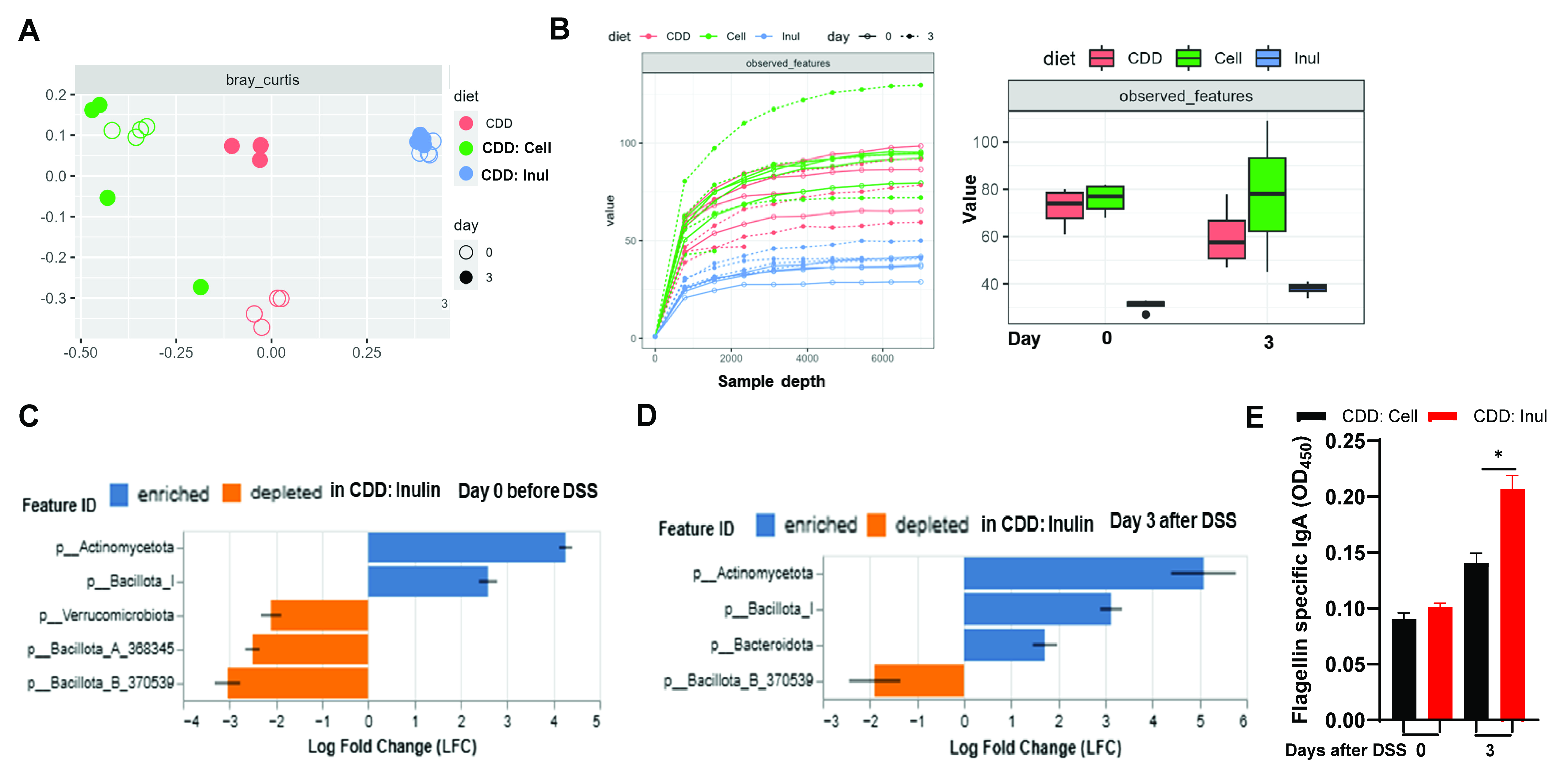

Supplement: Supplementary material — Figure S3. [file KGMI_A_2570425_SM4479.tif]
